# Phase-Specific Biomechanical Characterization of Upper Limb Movements in Stroke

**DOI:** 10.3390/bioengineering12111144

**Published:** 2025-10-23

**Authors:** Lei Li, Wei Peng, Jingcheng Chen, Shaoming Sun, Junhong Wang

**Affiliations:** 1 Hefei Institutes of Physical Science, Chinese Academy of Sciences, Hefei 230031, China; lleiww@mail.ustc.edu.cn (L.L.); wpeng@iim.ac.cn (W.P.); 2 University of Science and Technology of China, Hefei 230026, China; 3 CAS Hefei Institute of Technology Innovation, Hefei 230088, China; cjc324@mail.ustc.cn; 4 Institute of Artificial Intelligence, Hefei Comprehensive National Science Center, Hefei 230026, China

**Keywords:** stroke, upper limb function, hand-to-mouth task, phase-specific, biomechanics, torque smoothness, mechanical work

## Abstract

Stroke often leads to persistent upper limb dysfunction that impairs activities of daily living, yet objective biomechanical indicators for precise assessment remain limited. This study aimed to characterize phase-specific impairments in energy output, torque stability, and muscle coordination during the hand-to-mouth (HTM) task and to explore their potential for improving rehabilitation evaluation. Motion data from 20 stroke patients and 20 healthy controls were recorded using wearable surface electromyography and inertial measurement unit systems. A musculoskeletal model was applied to calculate joint torque, mechanical work, torque smoothness, and a novel torque-based co-contraction index across four movement subphases. These phase-specific metrics demonstrated significant correlations with clinical motor impairment scores, confirming their clinical validity. Significant dynamic features were then selected to construct machine learning models for group classification. Stroke patients showed reduced output capacity, increased torque fluctuations, and abnormal co-contraction patterns that varied across subphases. Among the classifiers, the quadratic support vector machine achieved the best performance, with an accuracy of 84.6% and an AUC of 0.853, surpassing models based on whole-task features. These findings demonstrate that phase-specific biomechanical features sensitively capture neuromuscular deficits in stroke survivors and highlight the potential of phase-specific biomechanics to inform future individualized rehabilitation assessment and treatment planning.

## 1. Introduction

Approximately 70% of stroke patients experience upper limb dysfunction characterized by spasticity and hemiparesis during the acute phase [1], with 40% persisting into the chronic phase [2]. These functional impairments significantly restrict patients’ ability to perform activities of daily living (ADLs), such as dressing, eating, and personal hygiene [1,2], substantially impacting their quality of life and social participation [3]. Analyzing ADLs has become an essential method for assessing upper limb functional recovery and setting rehabilitation goals in stroke rehabilitation research [4,5,6,7]. Among various ADLs, proximal upper limb coordination and control tasks such as hand-to-mouth, finger-to-nose, or lightweight object transfer have become classic paradigms for kinematic and kinetic analysis due to their prevalence, standardization, and representativeness in daily life [8,9,10,11]. The Second Stroke Recovery and Rehabilitation Roundtable (SRRR) in 2017 [12] also recommended analyzing such tasks to evaluate the quality of upper limb movements post-stroke and optimize rehabilitation protocols.

Studies indicate that post-stroke motor control disorders primarily arise from integrated deficits in strength, coordination, and segmental control [13,14]. The inability to generate and regulate muscle force fundamentally underlies persistent upper limb motor impairment months after stroke [14,15]. Arene et al. [16] found that central nervous system lesions post-stroke induce physiological changes in hemiparetic muscles and their motor units, accompanied by active or passive inhibition of antagonist muscles and abnormal muscle activation patterns, collectively impairing force generation capacity. Furthermore, alterations in muscle structure and mechanical properties [17], as well as joint and connective tissue remodeling [18,19], increase passive tension, further restricting voluntary torque generation [16]. Thus, a thorough examination of joint and muscle mechanical dynamics during daily living tasks execution in stroke patients helps elucidate personalized neuromuscular deficits and strength impairment patterns, providing a foundation for precision rehabilitation.

Currently, clinical assessments of joint and muscle function in stroke patients predominantly rely on isokinetic dynamometers or handheld isometric devices [1,20,21], methods limited by specific equipment and settings, thereby failing to capture natural ADLs biomechanics. Conversely, inverse dynamics methods integrating wearable sensors and musculoskeletal modeling offer effective solutions to these limitations [22,23], enabling analysis of joint torques, muscle forces, and their dynamics during daily activities outside laboratory conditions [23]. Shah et al. [24] proposed a virtual biomechanical shoulder robot model that conducts a detailed analysis of the complex kinematics of the human shoulder. Cop et al. [25] integrated system identification with biomechanical models to propose a closed-loop framework decomposing joint stiffness contributions into muscle/tendon components. Radmilović et al. [26] combined Hill-type muscle modeling to develop an elbow joint stiffness scale, observing an exponential stiffness increase with movement velocity and load. Tahmid et al. [27] focused on upper limb joint torque estimation via EMG-driven modeling, quantifying muscle dysfunction in stroke patients. Seth et al. [28] developed a shoulder musculoskeletal model to analyze muscle contributions during upper limb tasks.

Functional movement primitives or movement subphases, such as reaching or transporting, are considered building blocks of movements, representing finer and relatively stable motion elements [29,30]. Significant differences exist in functional requirements and neuromuscular control strategies among these subphases. Decomposing daily living tasks performed by stroke-affected individuals into fundamental primitives and assessing detailed joint and muscle mechanical characteristics during each subphase can reveal pathophysiological deficits and provide precise, clinically meaningful individualized impairment information [29]. Repnik et al. [31] quantified differences in five kinematic parameters across subphases of upper limb tasks in stroke patients, demonstrating the potential of motion quantification in differentiating participant groups. Kim et al. [32] identified task-related kinematic differences during drinking activities in hemiplegic subjects, highlighting specific kinematic patterns as promising clinical outcome indicators. However, existing studies predominantly focus on kinematic analyses, lacking in-depth exploration of muscle force and kinetic characteristics across task subphases.

This study utilized wearable surface electromyography (sEMG) and inertial measurement unit (IMU) systems to collect motion data from stroke patients and healthy controls performing a standardized hand-to-mouth (HTM) task. Joint torque characteristics of the shoulder and elbow, as well as major muscles, were computed using inverse dynamics and static optimization. The task was further segmented into four consecutive movement subphases, and the smoothness, mechanical work within each phase, and a novel Torque-based Co-Contraction Index (TCCI) were evaluated. Machine learning classification models, constructed based on phase-specific biomechanical features, significantly improved identification accuracy of stroke patients, validating the enhanced effectiveness of phase-specific quantification strategies and providing new insights for precision rehabilitation.

## 2. Materials and Methods

### 2.1. Participants

This study included two participant groups: 20 chronic-phase stroke patients exhibiting unilateral upper-limb impairments and 20 healthy individuals with no history of upper-limb or neurological disorders. Inclusion criteria included (1) first-ever unilateral stroke confirmed by neuroimaging with residual upper-limb motor impairment; (2) at least 30° of active elbow flexion and FM-UE > 20 to ensure sufficient motor capacity; (3) ability to maintain a seated posture and follow verbal instructions; and (4) adequate cognitive function (MMSE ≥ 24). Exclusion criteria included (1) recurrent or bilateral stroke, (2) severe spasticity (MAS > 2), (3) musculoskeletal deformity or pain limiting movement, (4) severe neglect, aphasia, or apraxia, and (5) cardiovascular or orthopedic conditions that might affect task safety.

### 2.2. Data Collection Procedure

A standardized Hand-to-Mouth (HTM) task [10], previously shown to have good test–retest reliability was employed to assess participants’ upper limb movement capacity during activities like drinking, eating, and approaching the face [11]. Participants simulated a drinking motion by reaching forward from an initial position, touching a marked point, raising their hand to touch their lips with the thumb, returning to the marked point, and finally resuming the initial position. The target point was set along the body’s midline, approximately 30 cm from the table edge, calibrated to 80% of individual arm length.

Participants sat on a height-adjustable chair with their backs supported but were allowed to adjust posture when necessary. The initial posture required a naturally downward upper arm, elbow flexed at approximately 90°, and the hand placed flat on the table, palm downward. Participants performed the task at a self-selected comfortable pace; stroke patients used the affected limb, while healthy participants used their dominant hand. Each participant performed five HTM trials, with the middle three trials averaged for subsequent analyses.

### 2.3. Data Recording

**Figure 1:**
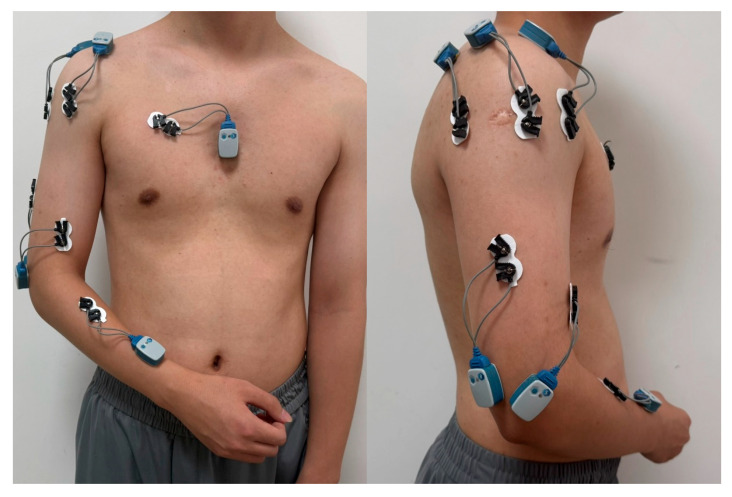
Wearable Sensor Configuration for Upper Limb Kinematics and Muscle Activity Monitoring.

Wearable sensors provided by Noraxon USA, Inc. (Scottsdale, AZ, USA) were used to record surface electromyography (sEMG) and inertial measurement unit (IMU) data. sEMG data were sampled at 2000 Hz from seven muscles: brachioradialis (BR), biceps brachii (BB), triceps brachii lateral head (TRL), anterior deltoid (AD), medial deltoid (MD), posterior deltoid (PD), and pectoralis major (PM). Bipolar Ag-AgCl electrodes (3M Healthcare Inc. Saint Paul, MN, USA) with a 20 mm inter-electrode distance were aligned parallel to muscle fibers and placed centrally on the muscle belly according to ISEA guidelines [33,34]. Skin preparation involved shaving and alcohol cleaning prior to electrode placement. IMU data were collected at 400 Hz from sensors attached to the sternum, upper arm, and wrist. All data were wirelessly transmitted via Bluetooth to a central computer for storage and processing. Sensor and electrode placements are illustrated in Figure 1.

### 2.4. Data Processing

Raw sEMG signals underwent band-pass filtering (30–450 Hz), full-wave rectification, and low-pass filtering (6 Hz) using a zero-phase, second-order Butterworth filter. sEMG envelopes were normalized to peak values recorded during task cycles and transformed into muscle activation $a_{m}\left(t\right)\in [0,1]$ using recursive filtering (cut-off frequency 2 Hz) and nonlinear transformation equations [35,36]. Synchronized IMU data underwent drift correction, temporal alignment, and attitude estimation [37,38]. Relative segment orientations were used to compute joint angles $\theta_{j}(t)(j=1-4)$, corresponding to shoulder flexion-extension (F/E), abduction-adduction (Ab/Ad), internal-external rotation (InR/ExR), and elbow flexion-extension (F/E).

Preprocessed EMG and kinematic data were imported into OpenSim (v4.3, Stanford University) [28] and analyzed using the Calibrated EMG-Informed Neuromusculoskeletal Modelling toolbox (CEINMS) [39]. Inverse kinematics (IK) was applied to obtain joint angles, and inverse dynamics (ID) was used to calculate net joint torques. CEINMS was then implemented in an EMG-informed mode: recorded EMG signals directly informed activations of measured muscles, while unmeasured muscles were estimated through optimization. A calibration procedure adjusted the following subject-specific parameters to minimize the error between predicted and experimental torques: tendon slack length, optimal fiber length, tendon stiffness, maximal isometric force, and activation dynamics parameters.

The Hill-type muscle model was used to calculate muscle force:

(1) $$F_{m}\left(t\right)=F_{0}^{m}\cdot \left(a_{m}\left(t\right)f_{l}\left(l_{m}\right)f_{v}\left(v_{m}\right)+f_{p}\left(l_{m}\right)\right)\cdot \cos\left(\phi \left(l_{m}\right)\right)$$

where $a_{m}\left(t\right)$ is muscle activation derived from EMG, $l_{m}$ is the fiber length, $v_{m}$ is the fiber contraction velocity, $f_{l}\left(l_{m}\right)$ and $f_{v}\left(v_{m}\right)$ are the normalized force-length and force-velocity relations, $f_{p}\left(l_{m}\right)$ is the passive force-length term, and $F_{0}^{m}$ is the maximal isometric force, ϕ is the pennation angle that changed with instantaneous fiber length.

The torque contribution of each muscle at a given joint was obtained as

(2) $$\tau_{m}=r_{m}\left(t\right)\cdot F_{m}\left(t\right)$$

where $r_{m}\left(t\right)$ is the instantaneous muscle moment arm. Net joint torque was computed as the sum of all contributing muscles:

(3) $$\tau_{joint}\left(t\right)=\sum \tau_{m}\left(t\right)$$

The EMG-informed approach ensures that abnormal activation patterns in stroke patients are preserved, while optimization supplements the contributions of unmeasured muscles. The resulting individualized muscle forces and joint torques were used to compute biomechanical metrics.

### 2.5. Time Series Signal Segmentation

**Figure 2:**
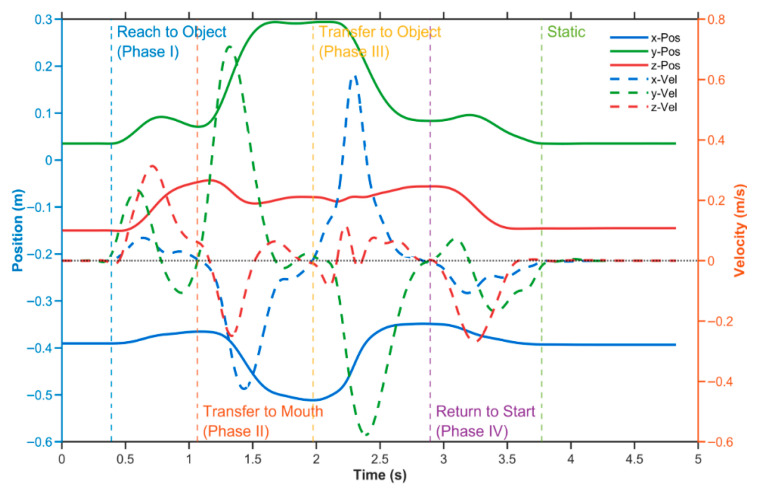
Phase-Segmented Kinematic Profiles During Standardized Hand-to-Mouth Task.

Based on functional modularity of upper-limb ADLs [10,11], the complete HTM movement sequence was segmented into four subphases: Reach to object phase (Phase I), Transfer to mouth phase (Phase II), Transfer to object phase (Phase III), and Return to start phase (Phase IV). A semi-automatic phase detection framework was employed, utilizing fused multi-dimensional motion trajectory features (position extrema and velocity changes) from the wrist-mounted IMU. Phase segmentation was defined as follows: Phase I onset was determined by the maximum displacement distance in the sagittal plane (x-direction) and the point where the velocity curve exceeded a preset threshold; Phase II was identified by the peak height in the vertical (gravity) direction (z-direction) combined with a velocity threshold; Phase III boundary was defined by the minimum x-direction position; Phase IV was detected based on velocity curve extrema (highest/lowest peaks) and a dynamic threshold of 2% absolute velocity. Automatic phase detection was verified by visual inspection and manually corrected if necessary. Kinematic trajectory characteristics for each phase are illustrated in Figure 2.

The accuracy of the semi-automatic phase segmentation was evaluated by comparing its output against a manually corrected reference standard. The mean absolute error (MAE) and root mean square error (RMSE) of all phase transition timings were calculated. The algorithm’s repeatability was assessed by executing it twice on the entire dataset. As a deterministic process, it produced identical outputs, confirmed by an intra-class correlation coefficient (ICC) for absolute agreement.

### 2.6. Feature Extraction

Musculoskeletal models enable movement scientists to examine muscle function by computing the mechanical work done by muscles during motor tasks [28,40]. To quantify the functional contribution of individual muscles during the HTM movement, muscle mechanical work was computed by integrating the time series of muscle power within each movement phase, based on kinetic parameters output by the musculoskeletal model.

Muscle power is defined as the product of the muscle-tendon unit force $F_{m}$ (computed via Equation (1)) and the fiber contraction velocity $v_{m}$ [28], $v_{m}$ is a core state variable in the Hill-type muscle model, solved in real-time during musculoskeletal simulation via muscle fiber dynamics equations. Positive power indicates concentric contraction; negative power indicates eccentric contraction. The mechanical Work for the $m_{th}$ muscle was computed as

(4) $$w_{m}=\int_{t_{m}}^{t_{m+k}}F_{m}\cdot v_{m}\;dt$$

where $\left[t_{m},t_{m+k}\right]$ correspond to the onset and termination of each subphase, $v_{m}$ is output synchronously by the musculoskeletal model during muscle dynamics solving (embedded within the force-generating function $F_{m}(a_{m},l_{m},v_{m})$).

Movement smoothness has been identified as a crucial indicator of motor recovery in stroke patients [41,42]. Spectral arc length (Sparc) is a frequency-domain metric, effectively quantifies smoothness of movements, forces, and impedances [43]. Based on muscle torque time-series signals ($\tau_{m}\left(t\right)=r_{m}\cdot F_{m}$, derived from Equation (2)), we applied Sparc to evaluate the dynamic smoothness characteristics of joint and individual muscle torques. This metric quantifies the stability and continuity of torque outputs by analyzing the spectral properties of the torque curves. Sparc values are negative, with values closer to zero indicating greater smoothness. Sparc was computed as follows [43]:

(5) $$Sparc=-\int_{0}^{\omega_{c}}\;\sqrt{{\left(\frac{1}{\omega_{c}}\right)}^{2}+{\left(\frac{d\hat{V}\left(\omega \right)}{d\omega }\right)}^{2}}d\omega$$

where $\hat{V}(\omega )$ is the Fourier magnitude spectrum of the normalized torque time-series, ω is the angular frequency (rad/s), and $\omega_{c}$ is an adaptively selected cutoff frequency.

The Co-Contraction Index (CCI)traditionally quantifies antagonist muscle co-activation levels during motor tasks using sEMG [44], providing insights into neuromuscular control strategies. However, the relationship between sEMG signals and actual muscle mechanical output is complex and highly nonlinear [45], limiting the ability to directly reflect biomechanical co-contraction effects. Therefore, based on muscle torque time-series from the musculoskeletal model $\tau_{m}\left(t\right)$, we proposed a torque-based Co-Contraction Index (TCCI). This approach quantifies the biomechanical synergy between agonist and antagonist muscles by integrating their overlapping torque signals, thus more effectively characterizing muscle co-contraction from a biomechanical perspective. TCCI was calculated as

(6) $$TCCI=\frac{1}{∆t}\int_{t_{m}}^{t_{m+k}}\;min\left(\tau_{agonist}\left(t\right),\tau_{\mathrm{antagonist}}\left(t\right)\right)dt$$

where $\tau_{agonist}\left(t\right),\tau_{\mathrm{antagonist}}\left(t\right)$ represent instantaneous torques of agonist and antagonist muscles computed via Equation (2). All torque values were normalized by the maximum torque observed throughout the whole task. $∆t=t_{m+k}-t_{m}$ denotes the duration of each phase. TCCI ranges between 0 and 1, where 0 indicates no overlap in muscle activation, and 1 represents simultaneous maximal activation throughout the whole phase. Higher TCCI values indicate increased joint stiffness through co-contraction, enhancing stability during movement.

### 2.7. Statistical Analysis

Smoothness and mechanical work values were calculated for seven muscles and four joint torques during each of the four task phases and the whole task. Additionally, TCCI values were computed for four muscle pairs across each subphase and the whole task: anterior–posterior deltoid (AD/PD), medial deltoid–pectoralis major (MD/PM), triceps brachii lateral head–biceps brachii (TRL/BB), and triceps brachii lateral head–brachioradialis (TRL/BR). Mean values from the middle three trials for each participant were utilized in statistical analysis, performed using SPSS 24.0 (IBM, Armonk, NY, USA). Shapiro–Wilk tests assessed normality for each variable. Independent-sample t-tests compared normally distributed continuous variables between stroke and healthy groups, while Mann–Whitney U tests were applied for non-normally distributed variables. Normally distributed variables were presented as mean ± standard deviation, and non-normally distributed variables as median and interquartile range. Statistical significance was set at a *p*-value < 0.05, with the False Discovery Rate (FDR) correction applied for multiple comparisons.

To assess the clinical relevance of the phase-specific biomechanical metrics, Spearman’s rank correlation analyses were performed between the Fugl-Meyer Upper Extremity (FMUE) score and a select subset of key metrics. These metrics were chosen based on their (a) demonstration of significant between-group differences, and (b) clear physiological interpretability. The selected metrics included elbow flexion-extension work, biceps brachii work, anterior deltoid work, smoothness of elbow flexion-extension and shoulder abduction-adduction torques, and TCCI of the AD/PD and TRL/BB muscle pairs. Correlation coefficients (ρ) are reported, and statistically significant correlations (*p* < 0.05 after FDR correction) are indicated with an asterisk (*) in the tables.

### 2.8. Machine Learning Algorithms and Modeling

Biomechanical indicators exhibiting significant differences (*p* < 0.05) between stroke and healthy groups (phase-specific and whole-task measures) were selected as input features for machine learning classification models, with labels defined as binary (stroke/healthy). Five-fold cross-validation was employed to avoid overfitting; the dataset was randomly partitioned into five mutually exclusive subsets, with each subset sequentially serving as the test set, while the remaining four subsets constituted the training set. This process was repeated for five iterations, ensuring each data point was used for both training and testing.

Five machine learning algorithms were utilized for classification: Quadratic Support Vector Machine (QSVM), Gaussian Support Vector Machine (GSVM), Logistic Regression (LR), Subspace Discriminant (SD), and Linear Discriminant (LD). QSVM maps data into a high-dimensional feature space using a quadratic polynomial kernel, establishing classification via maximum-margin hyperplanes [46]. GSVM applies a Gaussian kernel function to project data into high-dimensional space, effectively capturing nonlinear data relationships [47]. Logistic regression, a linear model, fits data using a log-odds function to estimate class probabilities [48]. Subspace discriminant analysis identifies optimal classification boundaries within lower-dimensional subspaces [49]. Linear discriminant analysis (LDA) seeks projection directions that maximize inter-class differences while minimizing intra-class variations [50].

Model performance was evaluated on independent test sets using accuracy, precision, recall, F1-score, and the Area Under the Receiver Operating Characteristic Curve (AUC), computed as follows:

(7) $$Precision=\frac{TP}{TP+FP}$$

(8) $$Recall=\frac{TP}{TP+FN}$$

(9) $$F1=2\times \frac{Precision\times Recall}{Precision+Recall}$$

where TP denotes true positives, FP false positives, and FN false negatives. All classification experiments were implemented using the Classification Learner toolbox in MATLAB R2023a.

## 3. Results

This study enrolled 20 chronic stroke patients and 20 healthy controls. No statistically significant differences were found between the groups regarding age, gender, or tested side distribution (*p* > 0.05). Detailed demographic characteristics are presented in Table 1.

The phase segmentation method demonstrated high temporal accuracy against the manual reference (MAE = 25.2 ± 12.8 ms; RMSE = 28.1 ms). The algorithm exhibited perfect test–retest reliability (ICC = 0.99), confirming its deterministic and repeatable nature.

Statistical analysis results (Table 2) revealed significant differences between stroke and healthy control groups across biomechanical metrics during each subphase and the whole Hand-to-Mouth (HTM) task.

In the mechanical work analysis, stroke patients exhibited significantly reduced elbow joint mechanical work during the Transfer to mouth phase (Phase II, *p* = 0.012) and the Transfer to object phase (Phase III, *p* = 0.009), indicating functional deficits in elbow torque generation. The anterior and middle deltoid bundles showed significantly lower work during the Reach to object phase (Phase I, *p* < 0.05). The Biceps Brachii displayed significantly lower work during both the Transfer to mouth phase (Phase II, *p* = 0.027) and the Transfer to object phase (Phase III, *p* = 0.028) compared to controls, reflecting reduced muscle output capacity. Notably, no significant differences were found between groups for joint mechanical work when analyzed over the whole task duration (*p* > 0.05).

Analysis of torque smoothness using the Spectral Arc Length (Sparc) metric demonstrated significantly degraded smoothness (more negative values indicating less smoothness) in the stroke group during specific task phases. During the Reach to object phase (Phase I), stroke patients demonstrated significantly worse Sparc values for elbow and shoulder flexion-extension, as well as for muscles including brachioradialis, biceps brachii, and the middle and posterior deltoid muscles (*p* < 0.05). In Phase II (Transfer to mouth), stroke patients showed significantly lower torque smoothness across elbow flexion-extension, shoulder abduction/adduction, internal/external rotation, and muscle torques (*p* < 0.05). In the Transfer to object phase (Phase III), torque smoothness deficits persisted in elbow flexion-extension torque, with significant differences noted specifically for the triceps brachii lateral head and anterior and middle deltoid muscles (*p* < 0.05). During the Return to start phase (Phase IV), Sparc values across all joints and muscles were significantly inferior in stroke patients compared to healthy controls, with the biceps brachii exhibiting the largest discrepancy (−2.60 ± 0.27 vs. −3.01 ± 0.37, *p* < 0.001). In contrast, when analyzing the whole HTM task, fewer significant differences were observed. Only the triceps brachii lateral head and posterior deltoid muscles showed significant group differences (*p* < 0.05).

Torque-based Co-Contraction Index (TCCI) results revealed significantly higher values in stroke patients for the anterior deltoid–posterior deltoid (AD/PD) and medial deltoid–pectoralis major (MD/PM) muscle pairs across all phases (*p* < 0.05). The triceps brachii lateral head–biceps brachii (TRL/BB) muscle pair showed no significant difference in Phase I (*p* > 0.05), while significant differences were found in other phases. The triceps brachii lateral head–brachioradialis (TRL/BR) muscle pair showed no significant inter-group differences in any subphase (*p* > 0.05). However, when analyzing the whole task, significant differences (*p* < 0.05) in TCCI values were observed for the anterior deltoid–posterior deltoid (AD/PD) and triceps brachii lateral head–brachioradialis (TRL/BR) muscle pairs.

**Figure 3:**
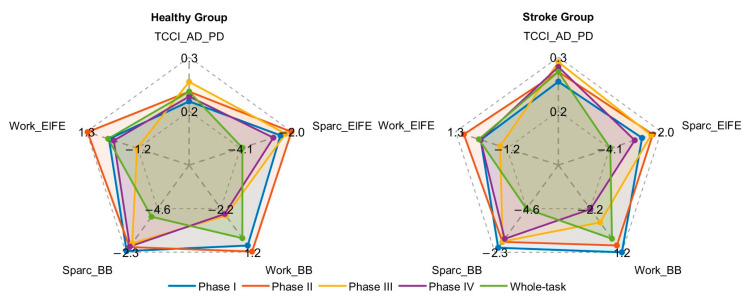
Radar plots of selected biomechanical metrics for healthy controls and stroke patients across movement phases.

To visualize the phase-specific differences outlined in Table 2, radar plots were generated for several key biomarkers that showed significant inter-group differences. Figure 3 displays distinct biomechanical profiles between stroke and healthy groups across the four movement phases.

Significant correlations were observed between the FMUE score and multiple phase-specific biomechanical metrics (Table 3). Notably, these associations were generally stronger for phase-specific metrics than for whole-task metrics. For instance, mechanical work output during functionally critical phases (e.g., elbow work in Phase II, ρ = 0.79) was strongly positively correlated with FMUE, whereas abnormal co-contraction (e.g., TCCI of AD/PD in Phase I, ρ = −0.88) was strongly negatively correlated. These findings substantiate that the proposed phase-specific biomechanical metrics capture clinically meaningful aspects of motor impairment after stroke.

Machine learning classification models trained on phase-specific biomechanical features effectively differentiated between groups. Results in Table 4 show that all models achieved higher classification performance when incorporating phase-specific features, outperforming models using only whole-task features (values in parentheses, Table 4). The Quadratic Support Vector Machine (QSVM) model performed best, achieving 84.6% accuracy and an AUC of 0.853, along with a precision of 93.3%, recall of 73.7%, and an F1-score of 82.4%. This was significantly better than using only full-task features (accuracy 76.9%). Other models, such as Subspace Discriminant (SD) and Linear Discriminant (LD), achieved 82.1% accuracy. The Gaussian SVM (GSVM) achieved 79.5% accuracy with phase-specific features, significantly higher than its 61.5% accuracy without phase segmentation.

## 4. Discussion

This study systematically quantified joint dynamics and muscle mechanical characteristics across four continuous subphases of a standardized hand-to-mouth (HTM) task performed by stroke patients by integrating wearable sensors and musculoskeletal modeling techniques. We analyzed post-stroke motor control deficits from three biomechanical dimensions: stability, coordination, and output capability of joint and muscle.

Regarding mechanical work, stroke patients generally demonstrated reduced mechanical work output during each subphase of the standardized HTM task compared to healthy controls. Table 2 indicates that elbow work significantly decreased during the Transfer to mouth phase (Phase II, 1.25 ± 0.48 vs. 0.90 ± 0.32, *p* = 0.012), while mechanical work decreased by 26% during the Transfer to object phase (Phase III, −1.16 ± 0.37 vs. −0.86 ± 0.32, *p* = 0.009). These results highlight a clear deficit in net mechanical work among stroke patients during both lifting and controlled lowering phases. Wong et al. [51] reported that “effort” or energy output itself is a major limiting factor for achieving movement goals during upper limb extension tasks, suggesting the observed reductions might reflect limited energy availability during critical functional phases in stroke patients. The analysis of muscle revealed this reduction in mechanical work reflects limited force and energy output capabilities of paretic muscles. Specifically, the brachioradialis, a primary elbow flexor-extensor muscle, showed mechanical work reductions of 42% (*p* = 0.027) and 32% (*p* = 0.028) during Phase II and Phase III, respectively, directly impairing joint mechanical efficiency. Chang et al. [52] similarly reported weakened muscle strength and abnormal force control in the spastic-paretic biceps brachii among chronic stroke patients, aligning with this study’s findings of significantly reduced mechanical work in the biceps brachii. Patten et al. [53] also emphasized that functional degeneration of upper limb muscles, including the biceps brachii, is a central manifestation of impaired force-generating capacity post-stroke. Although the overall mechanical work of the shoulder joint showed no significant inter-group differences (*p* > 0.05), the anterior deltoid muscle—a primary contributor—experienced a 75% reduction in mechanical work during the Reach to object phase (Phase I, 0.08 ± 0.07 vs. 0.02 ± 0.08, *p* = 0.031), and the pectoralis major exhibited a 50% reduction during the Return to start phase (Phase IV, *p* = 0.036). These observations suggest that when proximal muscle output was limited, stroke patients might compensate through scapular or trunk movements to maintain overall mechanical output. This finding aligns with previous research [9] indicating decreased shoulder muscle activation and impaired motor unit recruitment post-stroke commonly resulting in compensatory trunk movements.

While mechanical work characterizes energy output capacity, the observed reduction in torque smoothness provides a direct kinematic manifestation of impaired dynamic stability in joint and muscle control. Dynamic stability of the neuromuscular system represents its capacity to maintain steady control, which is directly manifested in the generation of smooth torque profiles for coordinated movement [54,55]. In contrast, the impaired stability following a stroke—resulting from discoordinated neural commands and faulty sensorimotor integration—directly precipitates fluctuations in joint torque. We quantified this manifestation of instability using the spectral arc length (Sparc), which captures the smoothness of the torque profile by calculating the arc length of the normalized Fourier spectrum [43]. This frequency-domain smoothness of torque can be viewed as an embodiment of the neuromuscular system’s optimization strategy to “minimize muscle tension variation” [56], reflecting the continuity and controllability of torque outputs. This study found continuously degraded smoothness of elbow flexion-extension torque in stroke patients across all four phases (*p* < 0.05), accompanied by noticeable torque fluctuations in related muscles (e.g., brachioradialis, biceps brachii, *p* < 0.05), reflecting neuromuscular control instability. Importantly, torque smoothness deficits were phase-specific: shoulder rotation and abduction-adduction smoothness both declined significantly during Phase II (*p* = 0.009, 0.012), with abduction-adduction also reduced during Phase IV (*p* = 0.001). Fluctuations in anterior deltoid torque were observed from Phase II to IV. These differences may stem from variations in neuromuscular control strategies across phases, such as insufficient feedforward activation, impaired postural transition abilities, or disrupted muscle synergies [29]. Overall, stroke patients exhibited substantial limitations in maintaining continuous and smooth muscle force adjustments, with torque outputs containing excessive fluctuations, impairing the continuity and stability of force control.

To further explore multi-muscle synergistic control characteristics in stroke patients, this study proposed a torque-based co-contraction index (TCCI), expanding traditional co-activation assessments based solely on sEMG signals. The results indicated significantly higher TCCI values for anterior–posterior deltoid and medial deltoid–pectoralis major muscle pairs in stroke patients across all HTM task subphases (overall *p* < 0.05), especially during phases demanding precise postural control, such as Reach to object phase (Phase I) and Return to start phase (Phase IV), suggesting increased co-activation across shoulder flexion-extension and abduction-adduction motions. Conversely, no significant differences were observed in elbow muscles (triceps brachii lateral head–brachioradialis) across any subphase (*p* > 0.05), implying preserved normal synergistic patterns at the elbow. These findings align with Beer et al. [57], who reported abnormal elbow muscle torque space tuning due to disrupted early activation patterns rather than isolated muscle weakness or spasticity, reflecting systematic central nervous deficits in torque regulation and internal model degradation. The elevated co-contraction we observed at the shoulder could similarly be attributed to a combination of impaired central motor commands, compensatory strategies for stability, and the presence of underlying spasticity, all contributing to the broader motor control deficit. Additionally, normal task-dependent flexor-extensor coordination in upper limbs is often replaced post-stroke by rigid co-activation patterns [58,59,60], explaining elevated shoulder TCCI values during Phases II and III.

Our findings substantiate the clinical relevance of the proposed metrics, as evidenced by their strong correlations with FMUE scores. The significant positive correlation for elbow work in Phase II (ρ = 0.79) highlights the functional importance of anti-gravity control [52] while the significant negative correlation for shoulder co-contraction in Phase I (ρ = −0.88) identifies it as a key biomarker of compensatory stabilization [9]. Importantly, the consistently stronger correlations of phase-specific over whole-task metrics compellingly positions the dynamic, phase-aware assessment as crucial for sensitive evaluation of motor deficits.

This research strongly support the value of a phase-specific analysis approach. Crucially, the phase segmentation method itself was demonstrated to be highly accurate and reliable, which validates its use for such detailed biomechanical analysis. This strategy proved effective in uncovering dynamic deficits that were obscured in the whole-task analysis. For instance, torque smoothness of elbow flexion-extension and shoulder flexion-extension motions showed no significant difference during the whole task. However, significant differences emerged for elbow flexion-extension during Phase I (*p* = 0.006) and Phase IV (*p* = 0.013), and for shoulder flexion-extension during Phase I (*p* = 0.007) and Phase IV (*p* = 0.003), illustrating that phase-specific assessment enhances sensitivity. Machine learning models supported this approach: the QSVM model’s accuracy improved from 76.9% without phase segmentation to 84.6% with phase-specific features, with an AUC increase of 0.09, demonstrating excellent performance in classifying stroke versus healthy states and verifying sensitivity and practicality of the musculoskeletal dynamic indicators proposed in this study.

The proposed framework can be directly translated into clinical practice in two key ways. First, it can serve as a bedside assessment tool, where a simplified sensor system provides therapists with a precise, phase-specific breakdown of a patient’s impairments (e.g., “poor shoulder coordination during lifting” or “unstable elbow control during return”). Second, these precise findings enable truly personalized rehabilitation, allowing therapists to design targeted exercises that address the specific deficits identified in each movement phase, moving beyond generic interventions.

This study has several limitations. Firstly, we acknowledge that the study’s focus on a chronic stroke cohort with a limited sample size restricts the generalizability of our results to acute and subacute patients. This design was necessary for the initial validation of our framework. Nonetheless, the methodology and biomarkers we have established provide a direct foundation for future investigations across all recovery stages. Secondly, the sample size, while adequate for the primary validation aims, precluded a stratified analysis by functional severity. However, the strong correlations observed between our biomechanical metrics and FMUE scores confirm their sensitivity to impairment levels. Future studies with larger cohorts are needed to definitively characterize distinct biomechanical profiles across severity subgroups. Thirdly, while the current segmentation method is robust, future iterations could seek to make the process fully automated. Furthermore, although our calibration process estimated subject-specific parameters such as tendon stiffness, these estimates could be further refined in future studies by incorporating direct measurements (e.g., via ultrasonography) to reduce reliance on optimization-based inference. Fourthly, potential confounding factors, such as compensatory trunk strategies and hand dominance, were not controlled for and may have influenced the interpretation of the limb-specific biomechanical results. Fifthly, the machine learning models have not yet been validated on an independent external dataset. While the internal validation results are promising, future work must include multi-center external validation to conclusively establish the models’ generalizability and clinical applicability. Finally, this study had a cross-sectional design, which limits the ability to assess the sensitivity of the proposed metrics to functional changes over time. A critical next step will be to conduct longitudinal studies to validate the sensitivity of these phase-specific biomarkers to rehabilitation-induced changes and their utility in tracking functional recovery.

## 5. Conclusions

This study segmented the hand-to-mouth task into four subphases, developing a phase-specific multidimensional biomechanical characterization framework. The system elucidated dynamic deficits in stability, synergistic control, and output capabilities in upper-limb movements of stroke patients systematically. Our findings demonstrate that a phase-specific analysis can overcome the limited sensitivity of whole-task analysis, revealing dynamic deficits that would otherwise be obscured. Machine learning classification models (QSVM) further validated the clinical utility of this framework, providing a novel technical pathway for quantifying functional impairments during activities of daily living. Future applications may include developing phase-customized feedback intervention systems and individualized precision rehabilitation frameworks targeting specific motor deficits, thus facilitating the translation of biomechanical assessment techniques into clinical practice.

## Figures and Tables

**Table 1:** The characteristics of participants.

Variable	Patient (20)	Healthy (20)
Age, median (IQR)	55 (12)	52 (13)
Gender (Male/Female)	12/8	10/10
Side of assessed (Right/Left)	18/2	17/3
Type of brain injury (Isch/Hemo)	13/7	/
Time since brain injury, months, median (IQR)	29.37 (22.48)	/
FMUE, median (IQR)	23.42 (17.19)	/

Abbreviation: FMUE, Fugl-Meyer Motor Assessment for the Upper Extremity; Isch, ischemic stroke; Hemo, hemorrhagic stroke.

**Table 2:** Biomechanical Metrics for Hand-to-Mouth Task in Healthy Controls and Stroke Survivors: Phase-Specific and Whole-Task Comparisons.

Metrics	Phase I			Phase II			Phase III			Phase IV			Whole-Task		
	Health	Stroke	*p*-Value	Health	Stroke	*p*-Value	Health	Stroke	*p*-Value	Health	Stroke	*p*-Value	Health	Stroke	*p*-Value
Work_ElFE	0.21 ± 0.38	0.08 ± 0.24	0.195	1.25 ± 0.48	0.90 ± 0.32	0.012 *	−1.16 ± 0.37	−0.86 ± 0.32	0.009 *	−0.03 ± 0.31	0.09 ± 0.29	0.22	0.25(0.16)	0.17(0.12)	0.191
Work_ShFE	0.38 ± 0.56	0.62 ± 0.58	0.204	0.13 ± 0.71	0.23 ± 0.98	0.709	−0.28 ± 0.71	−0.06 ± 0.50	0.28	−0.05(0.74)	−0.50(0.72)	0.062	0.18 ± 0.34	0.29 ± 0.40	0.354
Work_ShAA	0.09(0.12)	0.09(0.21)	0.643	0.09 ± 0.14	0.06 ± 0.15	0.43	−0.08 ± 0.09	−0.04 ± 0.11	0.266	−0.14 ± 0.14	−0.20 ± 0.16	0.233	0.01(0.10)	−0.05(0.13)	0.191
Work_ShERIR	−0.18 ± 0.10	−0.18 ± 0.10	0.863	0.02(0.14)	−0.00(0.20)	0.966	−0.01(0.12)	−0.02(0.10)	0.811	0.20(0.18)	0.17(0.12)	0.684	0.01(0.06)	0.00(0.12)	0.546
Work_BR	0.35(0.46)	0.04(0.31)	0.1	1.49 ± 1.53	1.61 ± 2.78	0.863	−1.39 ± 1.41	−0.53 ± 1.72	0.095	−0.05 ± 0.39	0.09 ± 0.32	0.243	0.13 ± 0.03	0.13 ± 0.03	0.807
Work_BB	0.71(2.38)	1.23(2.48)	0.361	1.18(1.00)	0.69(0.58)	0.027 *	−1.64 ± 0.84	−1.12 ± 0.53	0.028 *	−1.78 ± 1.95	−2.16 ± 1.71	0.524	0.13 ± 0.26	0.16 ± 0.26	0.69
Work_TRL	0.30(1.67)	0.46(2.11)	0.255	0.17 ± 1.32	0.06 ± 1.13	0.795	−0.15 ± 1.57	0.06 ± 0.95	0.626	0.03(1.35)	−0.58(1.18)	0.066	−0.41(0.64)	−0.51(1.08)	0.173
Work_AD	0.08 ± 0.07	0.02 ± 0.08	0.031 *	0.03(0.13)	0.00(0.09)	0.747	−0.01 ± 0.09	−0.00 ± 0.09	0.696	−0.11 ± 0.08	−0.08 ± 0.09	0.268	0.02(1.18)	0.34(1.59)	0.201
Work_MD	−1.52 ± 1.13	−0.81 ± 0.81	0.032 *	−0.09 ± 1.42	0.22 ± 1.09	0.455	0.06 ± 1.32	−0.36 ± 1.17	0.299	1.65 ± 1.10	1.15 ± 1.16	0.175	0.20(0.80)	0.33(0.80)	0.361
Work_PD	−0.09(0.25)	−0.19(0.60)	0.173	−0.00(0.32)	0.04(0.75)	1	0.03 ± 0.45	−0.01 ± 0.48	0.805	0.06(0.20)	0.24(0.36)	0.045 *	−0.04 ± 0.21	−0.10 ± 0.30	0.419
Work_PM	0.09 ± 0.44	0.29 ± 0.47	0.178	−0.31(0.66)	−0.34(0.77)	0.877	0.14 ± 0.61	0.22 ± 0.50	0.676	0.19(0.44)	−0.19(0.38)	0.036 *	−0.04(0.12)	−0.04(0.09)	0.684
Sparc_ElFE	−2.46(0.42)	−2.78(0.37)	0.006 *	−2.01 ± 0.25	−2.34 ± 0.44	0.007 *	−2.25 ± 0.12	−2.41 ± 0.29	0.029 *	−2.75 ± 0.28	−3.08 ± 0.43	0.007 *	−4.02 ± 0.51	−4.08 ± 0.41	0.699
Sparc_ShFE	−2.56(0.24)	−2.72(0.26)	0.013 *	−2.56 ± 0.26	−2.65 ± 0.30	0.325	−2.60 ± 0.16	−2.66 ± 0.23	0.293	−2.65 ± 0.27	−2.97 ± 0.37	0.003 *	−3.56 ± 0.39	−3.68 ± 0.36	0.324
Sparc_ShAA	−2.37(0.38)	−2.46(0.36)	0.211	−2.51 ± 0.18	−2.73 ± 0.32	0.012 *	−2.61(0.17)	−2.68(0.39)	0.361	−2.46(0.55)	−2.82(0.51)	0.001 *	−3.27 ± 0.31	−3.45 ± 0.33	0.091
Sparc_ShERIR	−2.29(0.33)	−2.36(0.31)	0.191	−2.41 ± 0.22	−2.82 ± 0.19	0.009 *	−2.42(0.23)	−2.51(0.19)	0.432	−2.34(0.42)	−2.92(0.21)	0.001 *	−3.17 ± 0.21	−3.37 ± 0.28	0.082
Sparc_BR	−2.49(0.13)	−2.64(0.28)	0.003 *	−2.55 ± 0.27	−2.66 ± 0.30	0.253	−2.54 ± 0.30	−2.65 ± 0.31	0.239	−2.66 ± 0.25	−2.95 ± 0.35	0.005 *	−3.82 ± 0.40	−3.95 ± 0.35	0.319
Sparc_BB	−2.36(0.17)	−2.54(0.31)	0.007 *	−2.57 ± 0.17	−2.85 ± 0.26	0.000 *	−2.77 ± 0.11	−2.87 ± 0.24	0.08	−2.60 ± 0.27	−3.01 ± 0.37	0.000 *	−4.17 ± 0.49	−4.59 ± 1.10	0.127
Sparc_TRL	−2.32 ± 0.16	−2.48 ± 0.47	0.166	−2.47 ± 0.15	−2.77 ± 0.28	0.000 *	−2.62 ± 0.14	−2.76 ± 0.24	0.029 *	−2.59 ± 0.27	−2.94 ± 0.35	0.001 *	−3.52(0.29)	−3.92(0.54)	0.009 *
Sparc_AD	−2.43(0.30)	−2.54(0.40)	0.319	−1.72 ± 0.13	−2.02 ± 0.29	0.000 *	−2.11 ± 0.14	−2.26 ± 0.26	0.036 *	−2.58 ± 0.34	−2.85 ± 0.41	0.032 *	−3.46(0.54)	−3.62(1.15)	0.201
Sparc_MD	−2.43(0.17)	−2.58(0.37)	0.022 *	−2.64 ± 0.16	−2.81 ± 0.26	0.015 *	−2.71 ± 0.14	−2.88 ± 0.27	0.017 *	−2.67 ± 0.25	−3.03 ± 0.39	0.001 *	−3.62 ± 0.21	−3.78 ± 0.33	0.076
Sparc_PD	−2.50(0.15)	−2.66(0.25)	0.006 *	−2.70 ± 0.19	−2.93 ± 0.25	0.003 *	−2.92 ± 0.12	−2.97 ± 0.24	0.446	−2.71 ± 0.25	−3.05 ± 0.33	0.001 *	−3.60 ± 0.14	−3.85 ± 0.21	0.000 *
Sparc_PM	−2.32(0.24)	−2.58(0.36)	0.013 *	−2.50 ± 0.18	−2.73 ± 0.34	0.011 *	−2.66(0.25)	−2.68(0.26)	0.164	−2.54 ± 0.25	−2.92 ± 0.39	0.001 *	−3.68(1.00)	−3.95(1.04)	0.966
TCCI_AD_PD	0.24 ± 0.03	0.28 ± 0.05	0.015 *	0.26(0.03)	0.30(0.05)	0.001 *	0.28 ± 0.03	0.32 ± 0.03	0.000 *	0.25(0.05)	0.31(0.06)	0.003 *	0.26 ± 0.02	0.30 ± 0.04	0.001 *
TCCI_TRL_BB	0.26 ± 0.04	0.28 ± 0.05	0.086	0.27 ± 0.02	0.31 ± 0.04	0.001 *	0.28 ± 0.03	0.32 ± 0.03	0.001 *	0.26(0.06)	0.31(0.05)	0.013 *	0.27 ± 0.03	0.30 ± 0.04	0.008 *
TCCI_MD_PM	0.30 ± 0.02	0.33 ± 0.03	0.000 *	0.30 ± 0.02	0.33 ± 0.03	0.000 *	0.30 ± 0.02	0.33 ± 0.03	0.000 *	0.30 ± 0.02	0.33 ± 0.03	0.000 *	0.13 ± 0.03	0.13 ± 0.03	0.807
TCCI_TRl_BR	0.14 ± 0.05	0.12 ± 0.04	0.15	0.17 ± 0.06	0.19 ± 0.04	0.364	0.12 ± 0.03	0.12 ± 0.03	0.837	0.09(0.03)	0.10(0.04)	0.527	0.30 ± 0.02	0.33 ± 0.03	0.162

Data were tested for normality using the Shapiro–Wilk test. Normally distributed variables are presented as mean ± standard deviation, while non-normally distributed variables are presented as median (interquartile range). * indicates statistical significance after False Discovery Rate (FDR) correction for multiple comparisons (*p* < 0.05). Sparc was used to assess torque smoothness of joints (ElFE: elbow flexion-extension; ShFE: shoulder flexion-extension; ShAA: shoulder abduction-adduction; ShERIR: shoulder internal/external rotation) and muscles (BR: brachioradialis; BB: biceps brachii; TRL: triceps brachii lateral head; AD/MD/PD: anterior/medial/posterior deltoid; PM: pectoralis major, sternal head). Sparc values closer to zero indicate smoother torque profiles. Work (J/kg) quantifies mechanical work. TCCI (torque-based co-contraction index, 0–1) is calculated as the integral overlap of agonist-antagonist torque, with higher values indicating greater joint stiffness. Movement subphases include: Phase I (Reach to object phase: reaching to touch the target), Phase II (Transfer to mouth phase: lifting hand to the lips), Phase III (Transfer to object phase: returning to the target), and Phase IV (Return to start phase: returning to the initial position).

**Table 3:** Spearman correlation coefficients (ρ) between selected biomechanical metrics and the Fugl-Meyer Upper Extremity score in stroke survivors.

Biomechanical Metric	Phase I	Phase II	Phase III	Phase IV	Whole Task
Elbow F/E Work	0.28	0.79 *	0.51	0.19	0.31
Shoulder F/E Work	0.22	0.48	0.45	0.15	0.26
Biceps Brachii Work	0.85 *	0.31	0.29	0.33	0.38
Elbow F/E Sparc	0.82 *	0.52	0.78 *	0.87 *	0.35
Shoulder Abd/Add Sparc	0.32	0.83 *	0.37	0.81 *	0.41
AD/PD TCCI	0.84 *	0.61	0.39	0.31	0.31
MD/PM TCCI	−0.88 *	−0.54	−0.82 *	−0.52	−0.55
TRL/BB TCCI	−0.29	−0.52	−0.48	−0.43	−0.46

* Correlation is significant at the *p* < 0.05 level.

**Table 4:** Performance of Machine Learning Classifiers in Differentiating Stroke Survivors from Healthy Controls.

Model	Accuracy	Precision	Recall	F1-Score	AUC
SD	0.821(**0.795**)	0.875(**0.867**)	0.737(0.684)	0.800(**0.765**)	0.837(**0.863**)
GSVM	0.795(0.615)	0.824(0.600)	0.737(0.632)	0.778(0.615)	0.815(0.761)
LR	0.795(0.744)	0.867(0.765)	0.684(0.684)	0.765(0.722)	0.840(0.850)
QSVM	**0.846**(0.769)	**0.933**(0.813)	0.737(0.684)	**0.824**(0.743)	0.853(0.763)
LD	0.821(0.718)	0.929(0.682)	0.684(**0.789**)	0.788(0.732)	**0.858**(0.775)

Values in parentheses indicate performance using features without phase segmentation. QSVM Quadratic SVM, GSVM Gaussian SVM, LR Logistic Regression, SD Subspace Discriminant, LD Linear Discriminant.

## Data Availability

The original contributions presented in this study are included in the article. Further inquiries can be directed to the corresponding author.
